# PTB-Associated Splicing Factor (PSF) Is a PPARγ-Binding Protein and Growth Regulator of Colon Cancer Cells

**DOI:** 10.1371/journal.pone.0058749

**Published:** 2013-03-13

**Authors:** Tamotsu Tsukahara, Hisao Haniu, Yoshikazu Matsuda

**Affiliations:** 1 Department of Integrative Physiology and Bio-System Control, Shinshu University School of Medicine, Asahi, Matsumoto, Nagano, Japan; 2 Department of Orthopaedic Surgery, Shinshu University School of Medicine, Asahi, Matsumoto, Nagano, Japan; 3 Clinical Pharmacology Educational Center, Nihon Pharmaceutical University, Ina-machi, Saitama, Japan; University of Saarland Medical School, Germany

## Abstract

Peroxisome proliferator-activated receptor gamma (PPARγ) is a nuclear receptor that plays an essential role in cell proliferation, apoptosis, and inflammation. It is over-expressed in many types of cancer, including colon, stomach, breast, and lung cancer, suggesting that regulation of PPARγ might affect cancer pathogenesis. Here, using a proteomic approach, we identify PTB-associated splicing factor (PSF) as a novel PPARγ-interacting protein and demonstrate that PSF is involved in several important regulatory steps of colon cancer cell proliferation. To investigate the relationship between PSF and PPARγ in colon cancer, we evaluated the effects of PSF expression in DLD-1 and HT-29 colon cancer cell lines, which express low and high levels of PPARγ, respectively PSF affected the ability of PPARγ to bind, and expression of PSF siRNA significantly suppressed the proliferation of colon cancer cells. Furthermore, PSF knockdown induced apoptosis via activation of caspase-3. Interestingly, DLD-1 cells were more susceptible to PSF knockdown-induced cell death than HT-29 cells. Our data suggest that PSF is an important regulator of cell death that plays critical roles in the survival and growth of colon cancer cells. The PSF-PPARγ axis may play a role in the control of colorectal carcinogenesis. Taken together, this study is the first to describe the effects of PSF on cell proliferation, tumor growth, and cell signaling associated with PPARγ.

## Introduction

Colon cancer continues to be a major public health problem. Worldwide, approximately 1 million new cases of colon cancer are diagnosed each year, with nearly 500,000 deaths attributed to this disease annually [1]. Most of these deaths occur as a consequence of late diagnosis. Although colon cancer develops in the colon and rectal tissues, the cancer cells can spread to other parts of the body, such as the liver, bone, brain, and lung, and form a new tumor. Because metastatic colon cancer is associated with high mortality [2]–[3], progression to metastasis is the critical point in colon cancer survival. Currently, chemotherapeutic agents are the main tools for treating colon cancer. However, most of these drugs are nonspecific or become less effective as tumor cells acquire multi-drug resistance. Therefore, novel therapeutic options are needed to reduce colon cancer mortality.

PPARγ is a member of the nuclear receptor super-family, whose members activate target gene transcription in a ligand-dependent manner [4]–[5]. Activation of PPARγ by thiazolidinediones (TZDs) leads to an altered metabolism in adipose tissue, skeletal muscle cells, and liver that collectively results in insulin sensitization [6]. PPARγ expression is increased in many types of cancer, including colon, lung, breast, and stomach cancer, suggesting that regulation of PPARγ might affect cancer pathogenesis [7], [8]. Although PPARγ is expressed at significant levels in human colon cancer cells and tissue [8], the role of PPARγ activation in colon cancer is still controversial [9]. Furthermore, the role of PPARγ activation in cancer in general remains unclear. A number of high affinity synthetic agonists exist for PPARγ, including rosiglitazone and troglitazone. It has been reported that these agonists inhibit the proliferation of a variety of human cancer cells. However, the mechanism of action in most cases points to receptor-independent effects [10]. Several studies describe the ability of a PPARα/γ agonist, TZD18, to induce glioblastoma cell toxicity in a receptor-independent manner [11]. This compound induced apoptosis through cell cycle arrest. The apoptotic events were mediated by down-regulation of Bcl-2, up-regulation of Bax, and activation of caspase-3. These results suggest that TZDs can induce apoptosis independent of PPARγ activation, primarily by activating the intrinsic apoptotic pathway.

PTB-associated splicing factor (PSF) is a multifunctional protein involved in transcription regulation, pre-mRNA processing, and DNA repair [12]. One of the most abundant nuclear proteins, it consists of a single polypeptide chain of ca. 76 kDa (determined by sodium dodecyl sulfate-polyacrylamide gel electrophoresis [SDS-PAGE]) [13]. The amino terminus is rich in proline and glutamine residues. PSF has multiple binding functions. A recent study revealed that PSF belongs to a family of putative tumor-suppressor proteins that contain an RNA-binding domain (RBD) and a DNA-binding domain (DBD) [14]. The DBD binds and represses the transcription of genes that have a PSF-binding site [14], [15]. Thus, PSF is a highly complex protein that may be an important component of the transcriptional repression of many different genes involving different mechanisms. Recently, Wang et al. reported that PSF has a central role in the reversible regulation of mammalian cell proliferation and tumorigenesis [16]. Alteration in the expression of PSF and its binding partners may have potential as a therapeutic strategy against cancer [17]. However, how the various activities of PSF are regulated in colon cancer cells is not yet clear. We hypothesized that PSF interacts with PPARγ. Therefore, the aim of the present study was to was to obtain evidence for a direct interaction between PSF and PPARγ in colon carcinogenesis. Our results showed that PPARγ interacts directly with PSF. To examine the PPARγ-dependent effects of PSF, we also compared the HT-29 cell line, in which PPARγ is highly expressed, with the DLD-1 cell line, in which PPARγ is poorly expressed, under PSF knockdown conditions. The differential proteomic patterns of the two cell lines were assessed by LC-MS/MS analysis. The level of PPARγ in colon tissue is equal to or greater than that in adipose tissue [18]. This observation suggests the special role of PPARγ in the colon, as reflected in part by the cell- or tissue-specific expression of the receptor [19]. The proteins differentially regulated in the two cell lines provide us with a better understanding of the events involved in colon cancer.

## Materials and Methods

### Materials

Mouse monoclonal anti-PSF antibody (sc-271796), rabbit polyclonal anti-PPARγ antibody (sc-7196), goat polyclonal anti-VDAC2 antibody (sc-32059), mouse monoclonal anti-β-actin antibody (sc-47778), PSF siRNA (sc-38304), and control siRNA (sc-37007) were purchased from Santa Cruz Biotechnology Inc. (Santa Cruz, CA, USA). Cyclic phosphatidic acid (cPA) and lysophosphatidic acid (LPA) were purchased from Avanti Polar Lipids Inc. (Alabaster, AL, USA). The CheckMate™/Flexi® Vector Mammalian Two-Hybrid System was purchased from Promega (Madison, WI, USA).

### Plasmid Construction

Full-length human PSF cDNA (GenBank™, BC051192) was purchased from IMAGE Clone Consortium (IMAGE number: 5262885). PCR primers were designed to include the entire open reading frame of PSF. *Kpn*I and *Xho*I overhangs were added in the sense and antisense primers, respectively. The sequence of the sense primer was: 5′-GTAAGGTACCATGTCTCGGGATCGGTTCCGGAGTCGTG-3′ (*Kpn*I site is underlined). The sequence of the antisense primer was: 5′-CACGCTCGAGCTAAAATCGGGGTTTTTTGTTTGGGCCTTCG-3′ (*Xho*I site is underlined). A 2124-bp PCR product was amplified using Tks Gflex™ DNA polymerase (Takara, Shiga, Japan), purified, digested with *Kpn*I/*Xho*I, and inserted into a pcDNA3.1 (+) vector (Invitrogen, Carlsbad, CA, USA). For the mammalian two-hybrid assay, full-length PSF and PPARγ1 (*Sgf*I/*Pme*I) were generated by PCR and cloned into the pFN11A or pFN10A vector of the CheckMate™/Flexi® Vector Mammalian Two-Hybrid System (Promega) at the *Sgf*I and *Pme*I sites. The sequence of the PSF sense primer was: 5′- CATAGCGATCGCCATGTCTCGGGATCGGTTCCGGAGTCGTG-3′ (*Sgf*I site is underlined). The sequence of the PSF antisense primer was: 5′- CGCGGTTTAAACCTAAAATCGGGGTTTTTT GTTTGGGCC-3′ (*Pme*I site is underlined). The sequence of the PPARγ sense primer was: 5′- CAGTGCGATCGCCATGACCATGGTTGACACAGAGATGCCATTC-3′ (*Sgf*I site is underlined). The sequence of the PPARγ antisense primer was: 5′- GCGCGTTTAAACCTAGTACAAGTCCTTGTAGATCTCCTG-3′ (*Pme*I site is underlined). The PSF deletion mutants (150–707, 290–707, 370–707, 450–707, and 662–707) were generous gifts from Dr. Xuesen Dong (University of British Columbia, Department of Urologic Sciences, British Columbia, Canada). All sequences were confirmed using a DNA analyzer (ABI model 3730xl) and BigDye® Terminator v3.1 Cycle Sequencing Kit (Applied Biosystems, Foster City, CA, USA).

### Cell Culture

The human colon cancer HT-29 cell line was obtained from American Type Culture Collection (Manassas, VS, USA). DLD-1 human adenocarcinoma cells were obtained from the Health Science Research Resources Bank (Osaka, Japan). Cells were grown in Dulbecco’s Modified Eagle’s Medium (DMEM; Nakarai Tesque, Kyoto, Japan) or RPMI 1640 medium (Nakarai Tesque) containing 10% (v/v) fetal bovine serum (FBS), 100 U/mL penicillin, 10 µg/mL streptomycin, and 2.5 µg/mL Plasmocin™ (Nakarai Tesuque) at 37°C in a humidified incubator with 5% CO_2_.

### Preparation of Subcellular Fractions

The NE-PER Cell Fractionation Kit (Pierce Biotechnology, Rockford, IL, USA) was used to isolate the nuclear fraction from cells, according to the manufacturer’s instructions. After the cytoplasmic fraction was separated, the nuclear fraction was subjected to brief centrifugation (1,000×*g*, 10 sec), and the interface was removed to reduce cytoplasmic contamination.

### Pull-down Assay with a Metal Affinity Resin

Hexahistidine (6×His)-tagged PPARγ fusion proteins or empty vector controls were expressed in BL-21 (DE3) cells. Transformed BL-21 cells were induced with 0.5 mM isopropyl-1-β-d-galactopyranoside (IPTG) (Invitrogen) for 12 h at 25°C and collected by centrifugation. Next, 1 mL of supernatant was incubated with 20 µL of the TALON metal affinity resin (Takara) at 4°C for 1 h in lysis buffer. The resin was washed 5 times with wash buffer (20 mM MES pH 7.4, 150 mM NaCl), and the proteins were eluted with 150 mM imidazole in wash buffer. The amount of PPARγ was quantified using the Protein Quantification Kit-Rapid (Dojindo, Kumamoto, Japan). For the pull-down assay, purified 6×His-tagged PPARγ (1 µg) was mixed with nuclear extracts from HT-29 cells in 50 µL of binding buffer containing 20 mM MES pH 7.4 and 150 mM NaCl; TALON resin was then added. After incubation for 2 h at 4°C, the resins were washed 5 times with 500 µL of wash buffer containing 20 mM MES pH 7.4 and 100 mM NaCl.

### In-gel Digestion and Protein Identification by MALDI-TOF MS

In-gel digestion of gel bands was performed as previously described [20]. Briefly, protein spots, which were excised from the gel, were de-stained with 100 mM ammonium bicarbonate in 50% acetonitrile. The gel pieces were dried and digested with sequencing-grade modified trypsin (Promega). The peptide solution was recovered, and residual peptides were extracted by shaking with 5% trifluoroacetic acid (TFA) in 50% acetonitrile. The combined solutions were concentrated using a lyophilizer. The tryptic peptides, which were dissolved in 0.1% TFA, were desalted with Zip-Tip (Millipore, Billerica, MA, USA) according to the manufacturer’s instructions, mixed with the equal volume of a matrix solution (10 mg/mL α-cyano-4-hydroxycinnamic acid in 50% acetonitrile/0.1% TFA), and applied to a target plate. MS/MS analyses were performed using the AB SCIEX TOF/TOF™ 5800 System (AB SCIEX, Foster City, CA, USA). Protein identification was performed through ProteinPilot™ software (AB Sciex, Framingham, MA, USA) using the UniProt database.

### Co-immunoprecipitation and Western Blot

HT-29 cells and DLD-1 cells were resuspended in lysis buffer containing 10 mM Tris-HCl (pH 7.4), 100 mM NaCl, 50 mM KCl, 0.05% Tween-20, 10% glycerol, and the Halt Protease Inhibitor Cocktail (Takara). After 15-min incubation on ice, the cell lysate was sonicated and centrifuged at 16,000×*g* for 10 min at 4°C. The supernatant was collected as the whole-cell extract. The cell lysate was pre-cleared by adding 5 µL of Protein A/G Plus-Agarose (sc-2003, Santa Cruz Biotechnology) and incubated for 1 h at 4°C. The mouse monoclonal anti-PSF antibody (200 µg/mL, sc-271796, Santa Cruz Biotechnology) and the cell extract were mixed and incubated at 4°C for 3 h. The sample was then mixed with 5 µL of IP matrix (ImmunoCruz™ IP/WB Optima B system, sc-45039, Santa Cruz Biotechnology) and incubated at 4°C overnight. After the incubation, the immunoprecipitates were washed 5 times with 0.5 mL of 10 mM Tris-HCl (pH 7.4), 0.5 M NaCl, 0.1 M KCl, and 0.025% Tween-20, and then eluted with SDS-PAGE reducing sample buffer. Samples were separated by 5–20% SDS-PAGE and western blotted. After washing, the membrane was incubated with a horseradish peroxidase-linked species-specific whole secondary antibody (anti-rabbit or -mouse IgG; GE Healthcare, Little Chalfont, UK) for 1 h at room temperature and then visualized with Pierce ECL Plus Western Blotting Substrate (Thermo Scientific, Pittsburgh, PA, USA) or EzWestLumi plus (ATTO, Tokyo, Japan).

### Quantitative Real-time PCR Analysis

Total RNA was prepared from HT-29 and DLD-1 cells using NucleoSpin® RNA II (Takara). Then, 0.5 µg of total RNA was used for the subsequent synthesis of cDNA using the ReverTra Ace qPCR RT Kit (Toyobo, Osaka, Japan) as recommended by the manufacturer. Quantification of mRNA levels was measured by using an ECO Real-Time PCR system (Illumina, Inc., San Diego, CA, USA) and SYBR Green Realtime PCR Master Mix -Plus- (Toyobo) with the following primer pair sets: PSF, 5′-TGCCATTCATGCTTCTATGCA-3′ (F) and 5′-GGCCTAGACACTCTCATGCTTTC-3′ (R); 18S rRNA, 5′-CAGCCACCCGAGATTGAGCA-3′ (F) and 5′-TAGTAGCGACGGGCGGTGTG-3′ (R). All PCRs were performed in a 10-µL volume using 48-well PCR plates (Illumina). The cycling conditions were 95°C for 10 min (polymerase activation), followed by 40 cycles of 95°C for 15 sec, 55°C for 15 sec, and 72°C for 30 sec. In order to determine which housekeeping genes were most suitable for the subsequent normalization of data, we initially selected 3 candidates: GAPDH, β-actin, and 18S-rRNA, commonly used internal controls in mammalian cells. After amplification, the samples were slowly heated from 55°C to 95°C with continuous reading of fluorescence to obtain a melting curve. The relative mRNA quantification was calculated by using the arithmetic formula 2^−ΔΔCq^, where ΔCq is the difference between the threshold cycle of a given target cDNA and an endogenous reference cDNA. Derivations of the formulas and validation tests have been described in Applied Biosystems User Bulletin No. 2.

### Small Interfering RNA

PSF expression was inhibited in HT-29 and DLD-1 cells by transfection with a small interfering RNA (siRNA) targeting PSF (Santa Cruz Biotechnology), using Lipofectamine RNAiMAX (Invitrogen). Cells were plated onto 6-well plates (Iwaki, Tokyo, Japan) at a density of 5×10^4^ cells per well in DMEM containing 10% FBS. Cells were transfected with 100 pmol/mL of mRNA-specific siRNA or scrambled control siRNA. The reduction in PSF levels was confirmed by western blot analysis.

### Measurement of Cell Proliferation

PSF was knocked down in HT-29 and DLD-1 cells, which were seeded in 96-well culture plates (5×10^3^ cells/well) and incubated for 24 h. Cell proliferation was determined using the Cell Counting Kit-8 (Dojindo, Kumamoto, Japan): 10 µL of Cell Counting Kit-8 solution was added to the medium and incubated for 2 h in an incubator with 5% CO_2_; the amount of orange formazan dye produced was calculated by measuring the absorbance at 450 nm in a microplate reader (Awareness Technology, Inc., Palm City, FL, USA).

### Detection of Cytoplasmic Vacuolization

DLD-1 and HT-29 cells were grown on 96-well plates in DMEM for 24, 48, and 72 h after transfection with PSF siRNA. At these time points, cells were examined under an Olympus fluorescent microscope. Images were analyzed by counting the total number of cells and the number of vacuolated cells.

PPARγ activation was determined in HT-29 or DLD-1 cells transfected with 125 ng of the pGL3-PPRE-acyl-CoA oxidase luciferase vector, 62.5 ng of the pcDNA3.1-PPARγ vector, and 12.5 ng of the pSV-β-galactosidase (Promega) vector, which were constructed as previously reported [21], [22]. Twenty-four hours after transfection, cells were treated with Opti-MEM (Invitrogen) containing the test compound dissolved in DMSO (up to 0.1%) and cultured for an additional 20 h. Luciferase activity was measured with the ONE-Glo Luciferase Assay System (Promega) using a LuMate microplate luminometer (Awareness Technology, Inc., Palm City, FL, USA).

### Mammalian Two-hybrid Assays

CV-1 cells were plated onto a 96-well plate (Iwaki) at a density of 1.5×10^4^ cells per well in DMEM containing 10% FBS. On the next day, cells were transiently transfected with 71 ng of the pGL4.31[*luc2P/GAL4UAS/Hygro*] vector, 14.3 ng of the pFN11A-PSF vector, 14.3 ng of the pFN10A-PPARγ vector, and 10 ng of the pSV-β-galactosidase vector (Promega) using the X-tremeGENE HP DNA transfection reagent (Roche Applied Science, Indianapolis, IN, USA). At 48 h after transfection, the cells were analyzed with the ONE-Glo™ Luciferase Assay System (Promega) using a Power Scan 4 microplate reader (DS Pharma Biomedical Co., Ltd.). Samples were run in quintuplets, and the mean ± SEM was calculated. Data are representative of at least 3 independent transfections.

### Statistical Analysis

Student’s *t*-test was used for statistical comparisons. Differences were considered significant when the P-value was below 0.05.

## Results

### Protein-protein Interactions Assessed by Pull-down Experiments

**Figure 1:**
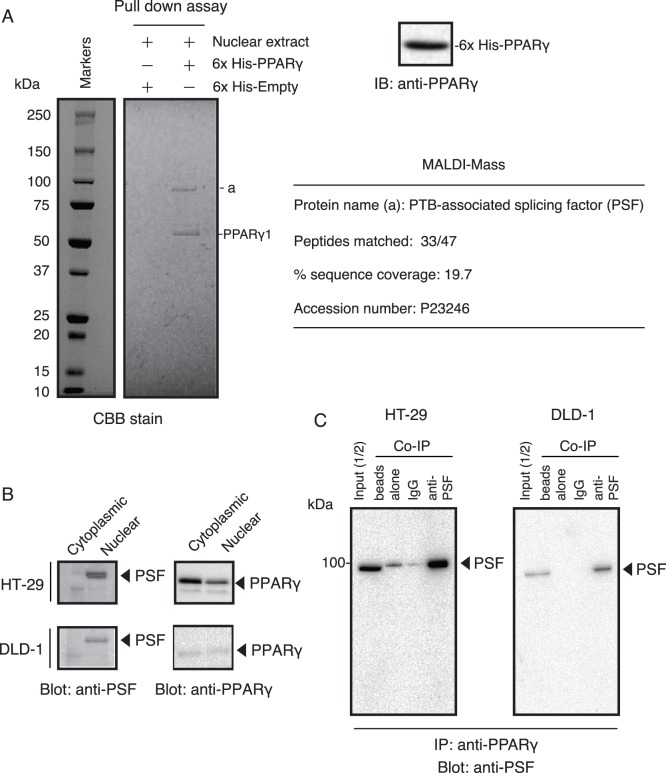
Physical interaction between PPARγ and PSF in HT-29 cells. (A) Pull-down affinity-binding assay with purified PPARγ. Full-length PPARγ expressed in *E. coli* as a 6×His-tagged fusion protein was isolated and purified using TALON resin (upper right panel). The 6×His-tagged PPARγ protein was incubated with nuclear extracts isolated from HT-29 cells. After washing with wash buffer, the resin was collected by centrifugation, and SDS-PAGE was performed with a 5–20% (w/v) acrylamide gel. The separated protein bands were visualized by Coomassie Brilliant Blue. The protein band (a) was excised from the gel, digested with trypsin, and identified by mass fingerprinting. The number of peptides, percentage of sequence coverage, and the accession number for the protein are given in Table S1. (B) Verification of the localization of PSF in nuclear and cytosolic extracts from HT-29 and DLD-1 cells. Cytosolic extracts and nuclear extracts were prepared from cells and analyzed by immunoblotting using an antibody against human PSF. Immunofluorescence staining of formalin-fixed HT-29 and DLD-1 cells shows the nuclear localization of PSF (right panel). (C) HT-29 cells were lysed with lysis buffer and then analyzed by co-immunoprecipitation and western blotting with anti-PSF antibody. Beads alone and normal rabbit serum (IgG) were used as negative controls. Arrows show the position of PSF (100 kDa).

Pull-down experiments with His-tagged fusion proteins attached to metal affinity beads are a screening technique for the identification of protein-protein interactions. Using the 6×His-tagged PPARγ as bait (Fig. 1A, right panel), we successfully captured a potential target protein (100 kDa) from HT-29 nuclear extracts (Fig. 1A). After extensive washing, bound proteins and the captured protein were excised from the gel, trypsin-digested, and analyzed by peptide mass fingerprinting with MALDI-MS. Tandem mass spectrometry (MS/MS) profiles identifying the PSF protein are shown in Fig. 1A. Because PSF is a nuclear protein, we then carried out cell fractionation, western blotting analysis, and immunostaining of PSF. As shown in Fig. 1B, in HT-29 and DLD-1 cell lines, PSF localized predominantly within the nuclear pellet. On the other hand, in HT-29 cells, PPARγ localized within the cytosolic and nuclear fractions. To further investigate the interaction between PPARγ and PSF, we performed co-immunoprecipitation (co-IP) experiments using nuclear extracts. As shown in Fig. 1C, PSF was detected with an anti-PSF antibody after immunoprecipitation of nuclear extracts from HT-29 and DLD-1 cells with an anti-PPARγ antibody. Thus, PSF and PPARγ interact within colon cancer cells.

### Interaction of pFN-PSF and pFN-PPARγ Fusion Proteins in CV-1 Cells

**Figure 2:**
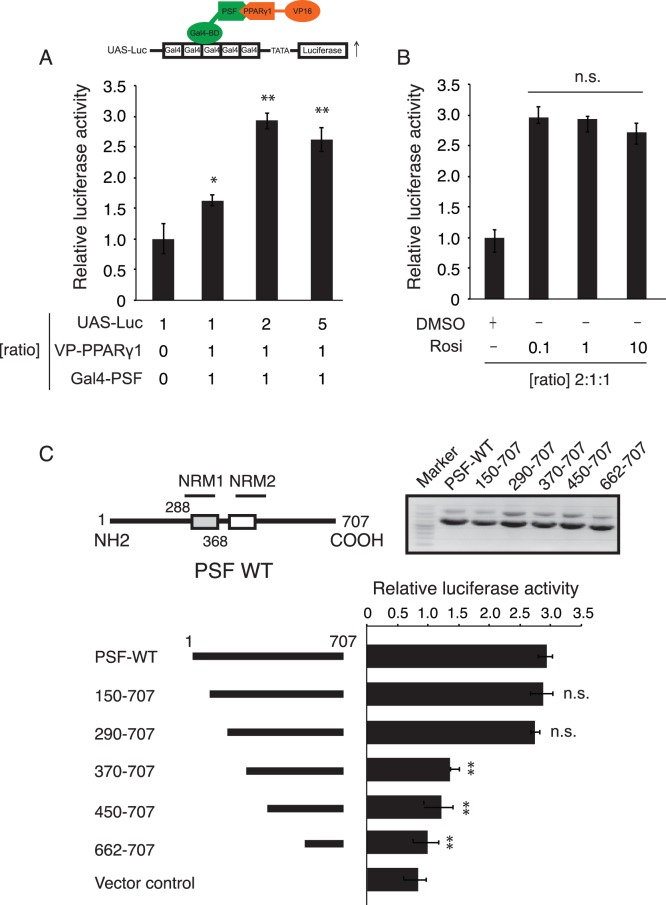
The first central region of the nucleotide-binding domain is required for the interaction with PPARγ. (A) Schematic diagram of the two-hybrid assay using full-length PSF and PPARγ. For the mammalian two-hybrid assay, CV-1 cells were co-transfected with GAL4-UAS-Luc alone or in combination with pFN11A-PSF (BIND) and pFN10A-PPARγ1 (VP16 transactivator). After 24 h of incubation, the cells were lysed, and luciferase activity was measured. The results are shown as fold induction compared to the negative control (GAL4-UAS-Luc alone) and represent the mean of triplicates from a representative experiment, with error bars showing the standard deviation. (B) CV-1 cells were co-transfected with GAL4-UAS-Luc, pFN11A-PSF, and pFN10A-PPARγ1. After 24 h of incubation, the cells were treated with rosiglitazone, and luciferase activity was measured. Rosiglitazone treatment (0.1–10 µM) did not affect the PSF-PPARγ interaction. (C) Schematic representation of PSF based on the domain prediction tool SMRT; the nucleotide-binding domain is indicated. Domains within PSF include the C-terminus nucleotide recognition motifs (NRM1 and NRM2) and the highly charged domain. The N-terminus contains proline-glutamine-rich domains and arginine-glycine-rich domains. The interaction of PPARγ1 with the truncated forms of PSF was analyzed using the mammalian two-hybrid assay. CV-1 cells were transfected with plasmids for the expression of the GAL4-UAS-Luc, pFN11A-PSF chimeric protein, VP-16-PPARγ1 proteins, and the indicated deletion mutants. The fold induction of luciferase activity was calculated relative to the negative control. The error bars represent the standard deviation.

To investigate the potential interaction between PSF and PPARγ, we analyzed their interaction in a mammalian two-hybrid assay in CV-1 cells. CV-1 cells were used because they do not express PPARγ [21]. As expected from previous experiments, co-expression of PSF, fused to the GAL4 DNA-binding domain, and PPARγ, fused to the VP16 activation domain, induced GAL4 promoter-driven luciferase expression (3.0-fold over that with empty vectors, Fig. 2A). The effect of rosiglitazone on the ability of PPARγ-PSF to induce luciferase expression was analyzed as shown in Fig. 2B. PPARγ activation did not significantly affect the PPARγ-PSF association. Next, we determined the physical location of the interaction sites. PSF is composed of 707 amino acids (aa), has a molecular mass of 76 kDa, and consists of 2 structural and functional domains [23]. In order to investigate which of these domains are crucial for the interaction with PPARγ, we constructed PSF deletion mutants. Interaction of chimeric Gal4-PSF deletion mutants with VP16-PPARγ was assessed using the mammalian two-hybrid reporter gene assay. As shown in Fig. 2C, loss of amino acids 1–290 of PSF had no effect on the interaction. Thus, the N-terminal domain is not essential for the interaction between these proteins. Loss of amino acids 291–370 of PSF disrupted the interaction between PSF and PPARγ. Deletion of amino acids 371–450, 451–662, and 452–707 of PSF also disrupted the interaction with PPARγ. Taken together, our results identified the first nucleotide binding domain (aa 291–370) as an important molecular site for PPARγ binding.

### PPARγ Activation does not Regulate PSF Expression in HT-29 and DLD-1 Cells

**Figure 3:**
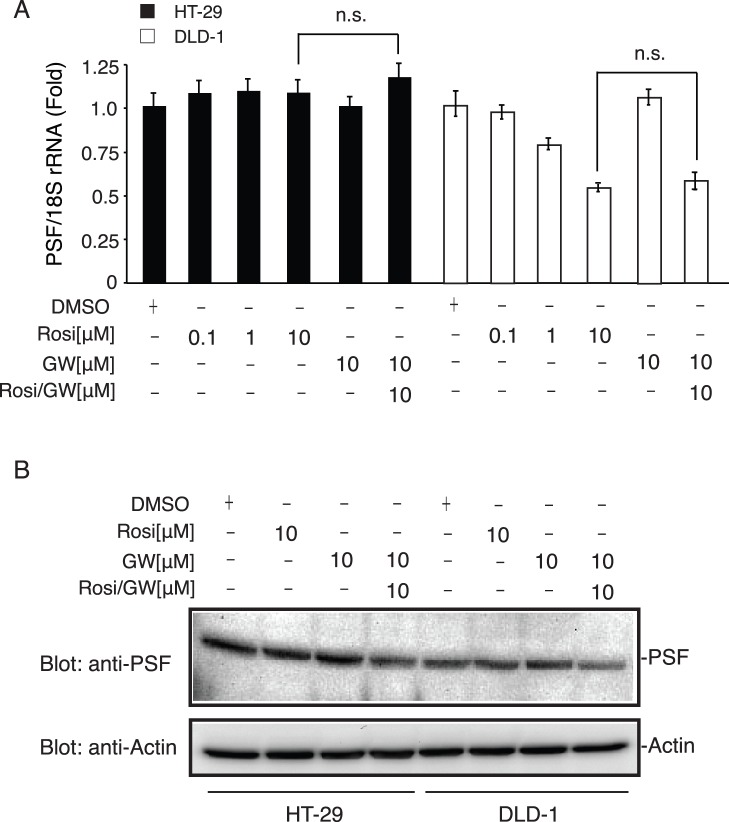
PPARγ activation is not involved in PSF downregulation in HT-29 and DLD-1 cells. (A) Real-time PCR measurement of PSF mRNA and protein expression in HT-29 and DLD-1 cells. Cells were treated with vehicle (DMSO), rosiglitazone (Rosi), or GW9662 (GW) for 20 h. PCR was performed using specific primers for PSF. The relative PSF levels were normalized to 18S- rRNA and are expressed as mean ± SEM (*n = *3), **P<0.01. The addition of GW9662 together with Rosi did not change PSF mRNA and protein expression levels. Protein levels were analyzed by SDS-PAGE and western blot and visualized with enhanced chemiluminescence reagent. Each lane was loaded with 50 µg whole-cell lysate. β-actin was used as a loading control.

To determine PPARγ’s role in regulating PSF expression, we examined the effect of a PPARγ agonist, rosiglitazone (10 µM), on PSF expression in DLD-1 and HT-29 cells. As shown in Figs. 3A and B, in HT-29 cells, stimulation with rosiglitazone did not inhibit PSF mRNA and protein expression; however, the expression levels decreased in DLD-1 cells stimulated with rosiglitazone. The selective and irreversible PPARγ antagonist GW9662 (10 µM) did not inhibit PSF expression in either cell line. Furthermore, addition of GW9662 and rosiglitazone did not change PSF mRNA and protein expression. These results suggest that PSF expression is PPARγ-independent and indicate that mechanisms other than PPARγ stimulation regulate the PPARγ-PSF axis.

### Knockdown of PSF Inhibits Cell Proliferation and Induces Vacuolation in DLD-1 Cells

**Figure 4:**
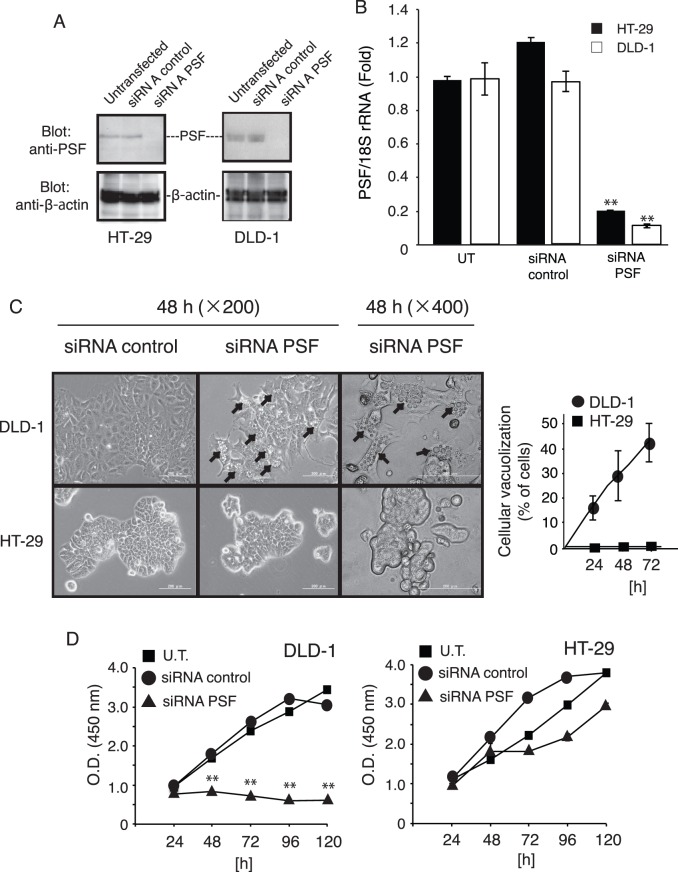
Downregulation of PSF inhibits the proliferation of colorectal cancer cells. (A) Expression of PSF was knocked down in HT-29 and DLD-1 cells. Total protein was extracted from untransfected (UT), control siRNA-, or PSF siRNA-transfected cells. Forty-eight hours later, whole-cell lysates were subjected to western blot analysis for PSF. Incubation with an anti-β-actin antibody was used as a protein-loading control. (B) The effect of siRNA on mRNA expression in HT-29 and DLD-1 cells. The efficiency of PSF knockdown was calculated to be 80% by real-time quantitative RT-PCR. Data are presented as means ± SEM (*n = *3). (C) At 24 h post transfection, cells were re-plated in 96-well plates (5×10^3^ cells/well) and incubated for 48 h. Cytoplasmic vacuolization was evident in DLD-1 cells in phase contrast images after siRNA transfection (indicated by arrows). Vacuolated cells were analyzed and counted as described in the Materials and Methods section. At least 3 fields of cells per sample were counted and tabulated. Data are expressed as mean ± SEM (*n = *3), **P<0.01. (D) Time-dependent cell growth inhibition was measured using the Cell Counting Kit-8 at 24, 48, 72, 96, and 120 h after siRNA transfection. An equal number of cells (1×10^5^ cells/well) were seeded in 6-well plates and then incubated for 24 h at 37°C in an incubator with 5% CO_2_. Then, 10 µL of Cell Counting Kit-8 was added to the medium and incubated for 2 h in the incubator (5% CO_2_). The amount of orange formazan dye generated was calculated by measuring the absorbance at 450 nm in a microplate reader. Data are expressed as means ± SEM (*n = *4), **P<0.01.

To evaluate the effects of PSF on the proliferation of HT-29 and DLD-1 cells, PSF expression was knocked-down using siRNA. As shown in Fig. 4A, knockdown of PSF expression in HT-29 and DLD-1 cells using siRNA was effective, as evidenced by western blot analysis using an anti-PSF antibody. As shown in Fig. 4B, real-time quantitative RT-PCR analysis showed that PSF mRNA in siRNA transfected cells was knocked down by 80–90% compared to expression in untransfected (UT) control cells. DLD-1 cells appeared as empty, lucent spaces in phase contrast images at 48 h after siRNA transfection (Fig. 4C). At 48 and 72 h after transfection, approximately 30 and 40% of the total number of cells, respectively, showed extensive vacuolization of the cytoplasm. Cell vacuolation increased in number and size, occupying increasingly larger areas of the cytoplasm in a time-dependent manner. Next, we determined the effect of PSF knockdown on cell proliferation by using a colorimetric assay. As shown in Fig. 4D, PSF knockdown severely inhibited cell proliferation in DLD-1 cells, which have a lower endogenous level of PPARγ than HT-29 cells. Interestingly, PSF knockdown weakly inhibited cellular proliferation in HT-29 and LOVO cells, compared to proliferation in DLD-1 and Caco-2 cells. Thus, HT-29 cells appear to be more resistant to PSF knockdown-induced growth inhibition.

### PPARγ Expression Level is Critical for Protection Against PSF Knockdown-induced Cell Growth Inhibition

**Figure 5:**
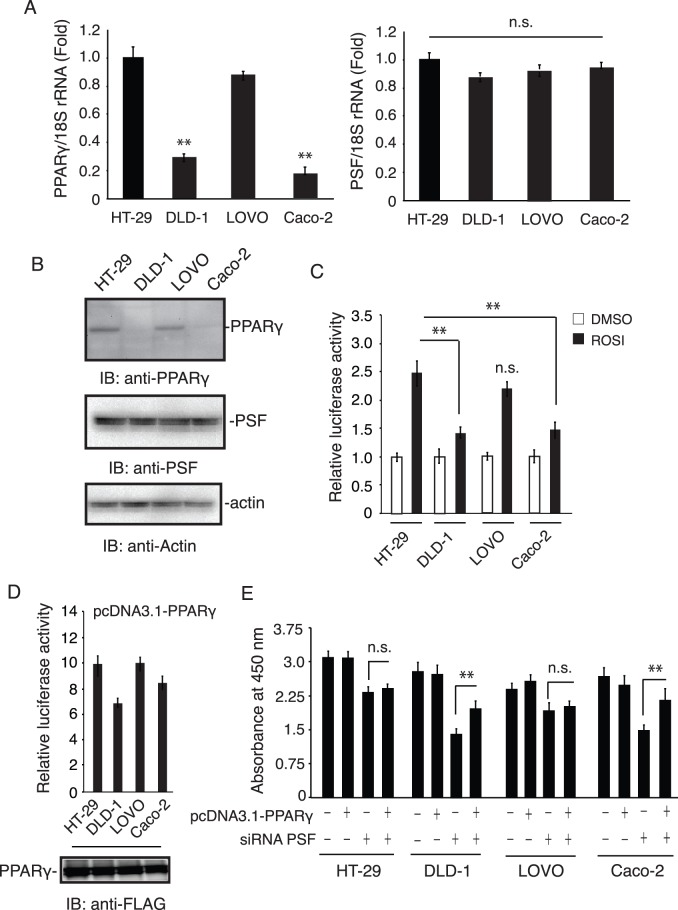
Real-time PCR measurement of PPARγ mRNA expression in 4 colon cancer cell lines. (A) The relative PPARγ levels (HT-29, DLD-1, Caco-2, and LOVO). normalized to 18S rRNA are expressed as mean ± SEM (*n = *3), **P<0.01. (B) Representative western blot of PPARγ1 expression. Cell lines were separated into nuclear and cytoplasmic fractions, and 50 µg of protein from the cytoplasmic fraction was analyzed by SDS-PAGE, western blotted, and visualized with enhanced chemiluminescence as described in the Materials and Methods section. (C) and (D) Effect of rosiglitazone on reporter activation in colon cancer cells. Cells were transiently transfected with a pGL3-PPRE-acyl-CoA oxidase luciferase reporter vector or pcDNA3.1-PPARγ vector. The cells were treated with 10 µM rosiglitazone for 20 h. Luciferase activity was normalized to *Renilla* luciferase activity. Data are expressed as mean ± SEM (*n = *4), **P<0.01. (E) Cells were transfected with expression plasmids encoding FLAG-PPARγ and siRNA PSF for 72 h. Next, 10 µL of Cell Counting Kit-8 was added to the medium and incubated for 2 h in an incubator with 5% CO_2_. The amount of orange formazan dye generated was calculated by measuring the absorbance at 450 nm in a microplate reader. Data are expressed as mean ± SEM (*n = *4), **P<0.01.

As shown in Figs. 5A and B, we investigated PPARγ and PSF mRNA and protein expression in 4 human colon cancer cell lines, HT-29, DLD-1, Caco-2, and LOVO. Total RNA was isolated from untreated cells. Real-time PCR analysis revealed that the relative level of PPARγ mRNA in these cells was in the order HT-29> LOVO>Caco-2> DLD-1. Similarly, our previous report suggested that the PPARγ protein level is high in HT-29 and LOVO cells and low in Caco-2 and DLD-1 cells [19]. This finding is also consistent with a report by Kitamura et al. [24]. Next, to test the functionality of PPARγ, we transfected the cell lines with a luciferase reporter plasmid. HT-29 and LOVO cells were more responsive to rosiglitazone than DLD-1 and Caco-2 cells (Fig. 5C). Because we observed an inverse correlation between the level of PPARγ expression and the sensitivity to PSF knockdown-induced inhibition of proliferation (see Fig. 4D), we reasoned that increasing the PPARγ expression level in transfected colon cancer cells with naturally low levels of PPARγ should reverse the PSF knockdown-induced effect on cell proliferation. To test this, we introduced the pcDNA3.1-FLAG-PPARγ plasmid (Fig. 5D) into the 4 human colon cancer cell lines 24 h after transfection with PSF siRNA. As shown in Fig. 5E, cell proliferation was increased by PSF knockdown, and this inhibitory effect was reversed by PPARγ overexpression. These data demonstrate that selective expression of PPARγ reverses PSF knockdown-dependent cell growth inhibition.

### Knockdown of PSF Expression by siRNA Induces Apoptosis in DLD-1 Cells

**Figure 6:**
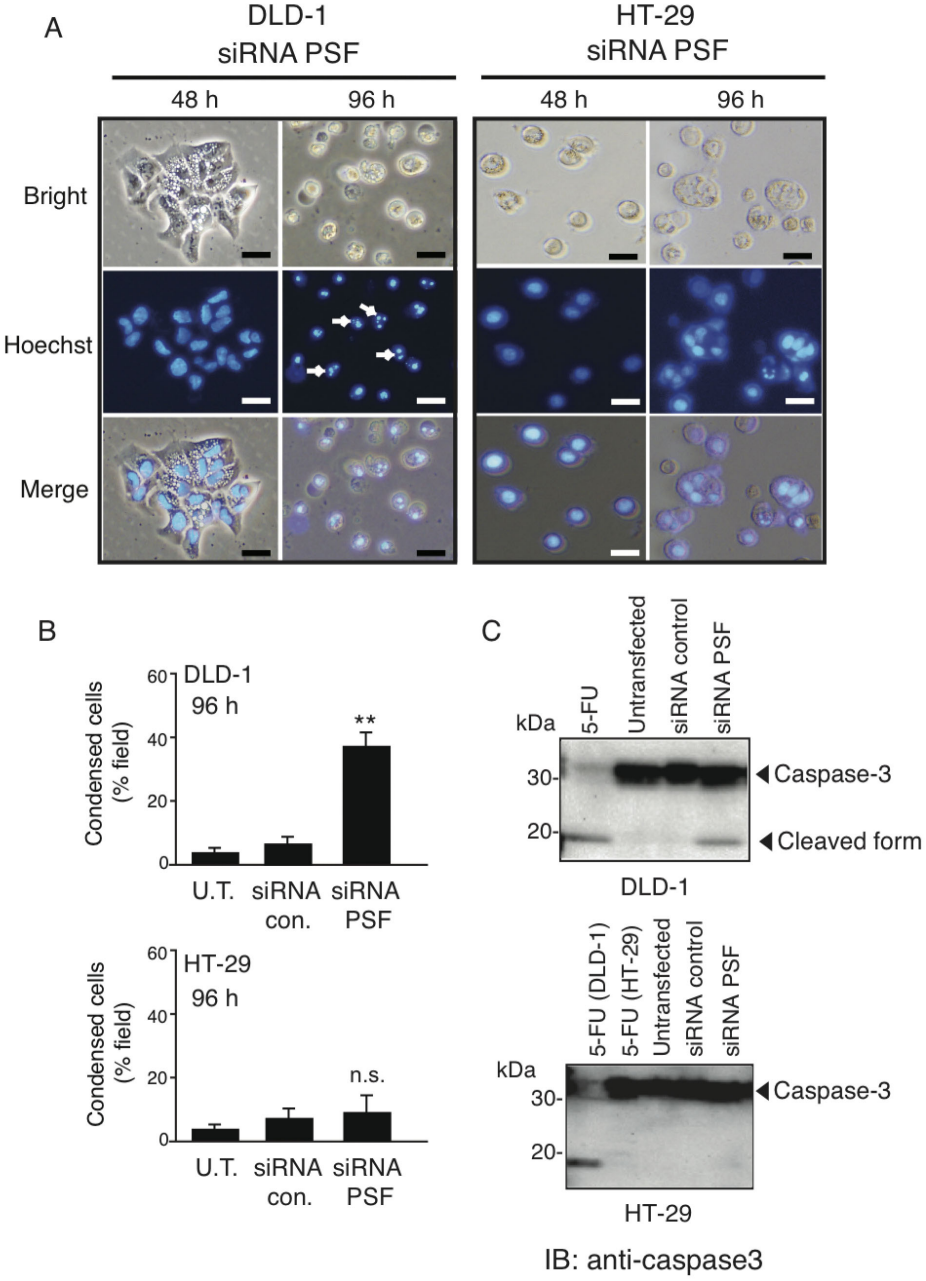
PSF knockdown induces DNA condensation in DLD-1 but not HT-29 cells. (A) Cells were seeded at a density of 5×10^3^ cells/well in 96-well plates in DMEM with 10% FBS. After 96 h, cells were stained with Hoechst 33342 and analyzed by fluorescence microscopy. Apoptotic nuclei were brightly stained compared to nuclei in untransfected cells or siRNA control-transfected cells. (B) At least 5 fields of cells per sample were counted and tabulated; values are expressed as mean ± SEM (*n* = 5), **P<0.05 based on Student’s *t*-test. (C) Both cell lines were seeded at a density of 1×10^5^ cells/well in 6-well plates in DMEM with 10% FBS. After 96 h, cell lysates were collected in RIPA buffer, and 50 µg of protein was loaded for SDS-PAGE. The apoptosis assay was carried out using an anti-caspase-3 antibody. 5-Fluorouracil (5-FU, 10 µM) was used as a positive control.

The decreased cell proliferation observed in conjunction with the morphological observations suggested that DLD-1 cells treated with PSF siRNA undergo apoptosis. To test this, cultures of DLD-1 and HT-29 cells were stained with Hoechst 33258 dye for 48 and 96 h. Hoechst 33258, a DNA sensitive fluorochrome, was used to assess changes in nuclear morphology following PSF knockdown. After knockdown of PSF for 96 h, DLD-1 but not HT-29 cells underwent morphologic changes typical of apoptosis, e.g., chromatin condensation and nuclear shrinkage (Figs. 6A and B). To verify the type of cell death induced by PSF knockdown, western blot analysis was performed to confirm that caspase-3 was activated by PSF knockdown. Caspase-3 has a key role in apoptosis, being responsible for the proteolytic cleavage of many key proteins [25]. Caspase-3 was primarily present in its 35-kDa pro-form (Fig. 6C) in untreated DLD-1 cells. Following 24 h exposure to 5-fluorouracil, which was used as a positive control, the p17 fragment of cleaved, active caspase-3 was detected. The p17 fragment was also detected after treatment with PSF siRNA for 96 h. These results indicate that PSF knockdown induces apoptosis in DLD-1 cells but not in HT-29 cells and that decreasing PSF expression in DLD-1 cells can inhibit cell proliferation.

### Protein Abundance Changes Upon PSF Knockdown

**Figure 7:**
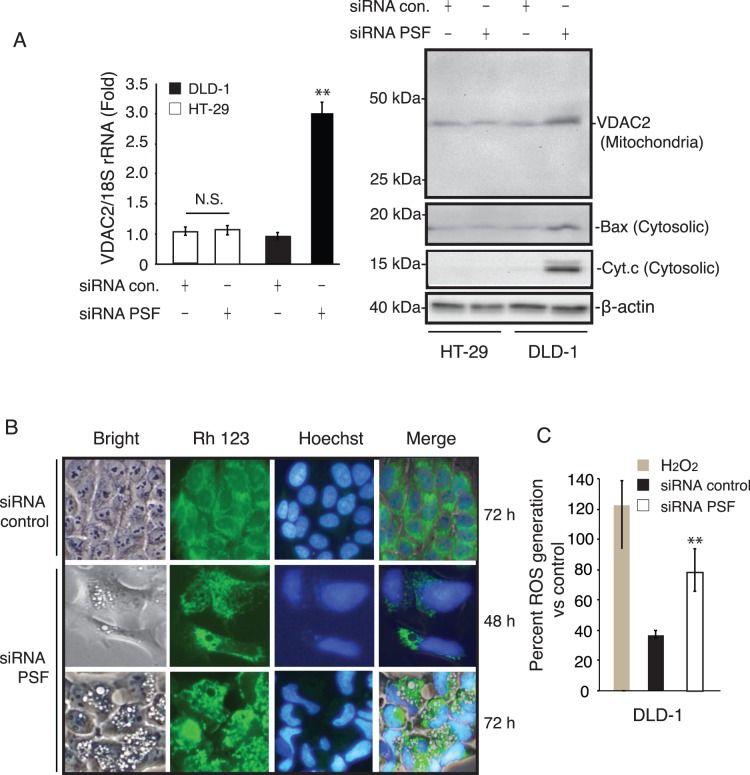
VDAC2 levels are up-regulated under PSF knockdown conditions in DLD-1 cells. (A) After PSF siRNA transfection, the expression of VDAC2 was further confirmed at the transcriptional level by real-time PCR and at the protein level by western blot. (B) Fluorescence microscopy of living cells stained with rhodamine 123. DLD-1 cells were stained with 100 nM rhodamine 123 for 30 min, rinsed in PBS, and imaged on an Olympus fluorescence microscope using a Cy3 filter. Nuclei were stained with Hoechst 33342. (C) ROS generation in DLD-1 cells after PSF knockdown. Intracellular production of ROS in DLD-1 cells treated with PSF siRNA. Hydrogen peroxide (100 µM) was used as positive control. Data are expressed as mean ± SEM (*n = *3), **P<0.01.

Next, we carried out a comparative proteomic analysis of proteins identified after PSF knockdown in DLD-1 and HT-29 cells. For this study, crude whole cell pellets were isolated from cells and lysed using the freeze-thaw method followed by Dounce homogenization and centrifugation (13,000×*g*, 20 min, 4°C). Using mass spectrometry and proteomics analysis, we identified 25 distinct proteins whose levels were significantly altered following PSF knockdown in both cell lines (Table S1). We then identified candidate proteins potentially involved in the PPARγ-PSF interaction and apoptosis. As expected from previous experiments (Figs. 4A–E), many of these proteins play a role in apoptosis and cell cycle regulation and act as molecular chaperones. Interestingly, in DLD-1 cells, voltage-dependent anion selective channel protein 2 (VDAC2) was up-regulated. We investigated VDAC2 mRNA and protein expression in DLD-1 cells after PSF knockdown. As shown in Fig. 7A, real-time PCR and western blot analysis confirmed that VDAC2 and Bax were upregulated under PSF knockdown conditions in DLD-1 cells. Next, we examined cells by fluorescence microscopy after staining with the mitochondria-specific dye rhodamine 123 [26] to determine whether there were changes in mitochondrial morphology after PSF knockdown. Cells were pre-incubated with rhodamine 123 for 30 min. Cells showed intense vacuolation after PSF knockdown (72 h), mostly in the perinuclear region (Fig. 7B). Large and medium size cells tended to be vacuolated. Vacuoles were never observed in mitochondria and nuclei. Next, we investigated whether PSF knockdown causes reactive oxygen species (ROS) formation in DLD-1 cells using 2′,7′-dichlorofluorescin diacetate (DCF) as a reporter of intracellular oxidant production. A DCF response was detected at 72 h post-transfection (Fig. 7C).

## Discussion

In the present study, we showed that PSF interacts with PPARγ in colon cancer cells. The interaction was originally revealed by using a mammalian two-hybrid assay and was subsequently confirmed in cell cultures by pull-down assays and co-immunoprecipitation experiments. PSF is a multifunctional protein that functions as transcriptional repressor for several nuclear receptors [27]. Increased expression of PSF in tumor cells suppresses tumorigenesis [14]. This finding suggests that PSF has a central role in the regulation of cell proliferation and tumorigenesis and therefore presents a potential therapeutic strategy for cancer. However, the function of PSF in regulating colon cancer cells has not been reported.

To date, a limited number of direct targets for PPARγ have been identified in studies using colon cancer cells. PPARγ has been found in cells from various lineages, e.g., colon cancer [19], stomach cancer [28], breast cancer [29], and prostate cancer [30]. PPARγ is recognized as a transcription factor that participates in the regulation of adipocyte differentiation. PPARγ agonists are currently in clinical use for the treatment of Type II diabetes [31]. While previous studies demonstrated that some PPARγ agonists inhibit the growth of cancer cells [32], many reports show that PPARγ ligand-mediated growth inhibition seems to vary depending on the cancer cell type. In colon cancer cells, the growth-suppressing effect of PPARγ ligands evident in *in vitro* studies was not clearly confirmed by *in vivo* studies [33]. Activation of PPARγ increases colonic polyps in the APC^+/min^ mouse model of colon carcinogenesis [34], [35]. These results may be due, in part, to PPARγ-dependent and -independent pathways.

The results of this study and those presented in a previous report [19] suggest that PPARγ overexpression in DLD-1 cells impedes cell growth inhibition. The effect on growth inhibition may depend on the quantity of PPARγ protein present and its interaction with PSF. In this study, our results showed that PPARγ mRNA and protein were expressed at various levels in 4 colon cancer cell lines. The effects of PSF expression in these cell lines varied, in a manner that correlated with the level of PPARγ expression. The proliferation of DLD-1 and Caco-2 cells, which express a low level of PPARγ, was significantly inhibited by knockdown of PSF, whereas the proliferation of HT-29 and LOVO cells, which express a higher level of PPARγ, was inhibited weakly or not at all. In DLD-1 cells, but not HT-29 cells, PSF knockdown also induced morphological changes associated with apoptosis, i.e., cell shrinkage and condensation of nuclear chromatin. Cornillon et al. reported that cells undergo extensive vacuolation as they proceed towards apoptosis [36]. Consistently, PSF knockdown did not induce vacuolation in HT-29 cells, whereas increased cell vacuolation was observed after PSF knockdown in DLD-1 cells. Thus, we observed distinct cell type-specific differences associated with the PPARγ-PSF interaction. In DLD-1 cells, but not HT-29 cells, PSF knockdown also induced morphological changes associated with apoptosis, i.e., cell shrinkage and condensation of nuclear chromatin. Furthermore, PSF knockdown did not induce vacuolation in HT-29 cells, whereas increased cell vacuolation was observed after PSF knockdown in DLD-1 cells. Thus, we observed distinct cell type-specific differences associated with the PPARγ-PSF interaction. Cornillon et al. reported that cells undergo extensive vacuolation as they proceed towards apoptosis [36]. We suggest that the process of cytoplasmic vacuolation can lead to a particular and distinctive form of cell death.

**Figure 8:**
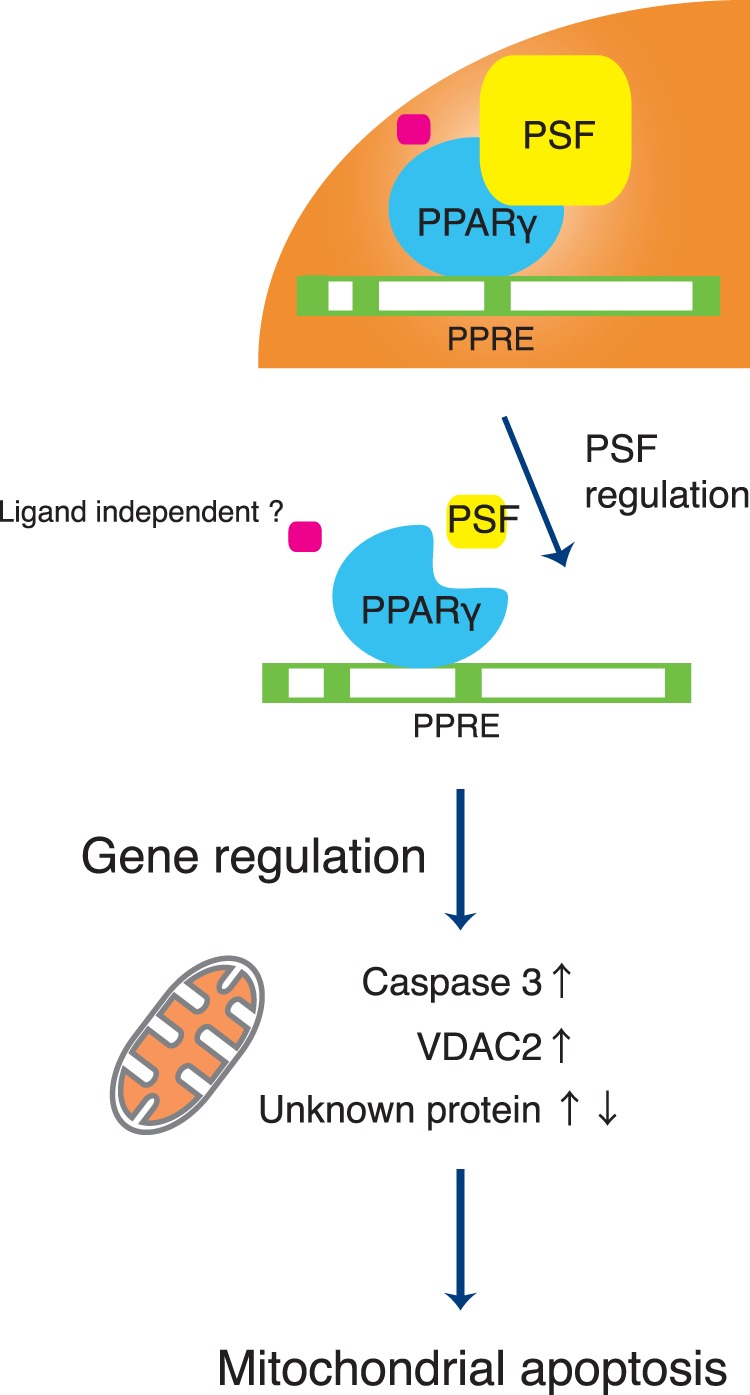
Scheme for the role of PSF in PPARγ-mediated gene regulation.

The main hypothesis driving this study is that activation of apoptosis plays a pivotal role in the PSF-PPARγ axis in colon cancer cells. During the induction of apoptosis, the permeability of the mitochondrial membrane changes, cytochrome c leaks into the cytoplasm, and caspases are activated [37]. Bax-induced caspase activation, via the release of cytochrome c from the mitochondria through VDAC, promotes the apoptosis pathway. Increased expression of mitochondrial VDAC and subcellular co-localization of VDAC/Bax increases mitochondrial permeability and apoptosis [38]. Thus, a particularly important outcome of the present study was the discovery that down-regulation of PSF stimulated apoptosis and markedly increased VDAC2 levels in DLD-1 cells. VDAC2 forms the pores of the outer mitochondrial membrane, and its involvement in mitochondrial-dependent apoptosis has been studied previously [39], [40]. It has been reported that VDAC2 normally inhibits the pro-apoptotic activity of Bak and that apoptotic signals induce the dissociation of Bak from VADC2 [41]. Chandra et al. suggested that an increase in VDAC2 complex formation in stimulated HCT116 colon cancer cells might be a pro-survival mechanism activated by apoptotic stimuli [41]. VDAC isoforms are also important regulators of mitochondrial metabolic activity, which is required for ROS production [42]. An extensive number of reports indicate that an increase in intracellular ROS production induces apoptosis through the mitochondrial pathway [43]. However, it is unclear whether ROS produced in the mitochondria are important in the regulation of cell death. Interestingly, in this study, we detected ROS production after PSF knockdown in DLD-1 cells. We propose that elevated levels of ROS may induce PSF-PPARγ signaling and regulate the apoptotic machinery. Although the mechanism of growth inhibition via the PPARγ-PSF axis in colon cancer cells has not been fully elucidated, our present study demonstrated that the PSF expression level is an important regulatory element for colon cancer cell growth. Understanding the diverse molecular interactions between PSF and it targets in the cancer system will provide insight into the pathogenesis of colon cancer. Therapies directed at PPARγ expression or its binding partners may lead to novel approaches to treat colon cancer. The effect of PSF on PPARγ targets and their contributions to PSF-mediated cellular processes requires further investigation (Fig. S1 and Fig. 8). PPARγ-interaction partners may provide insight into the biological functions of PPARγ and provide us with a better understanding of the events involved in colon cancer.

## Supporting Information

Figure S1 ***P21*** **mRNA levels were down-regulated under PSF knockdown conditions in DLD-1 and Caco-2 cells.** (A) After PSF siRNA transfection, the expression of *P21* mRNA was further confirmed at the transcriptional level by real-time PCR. The relative PPARγ levels normalized to 18S rRNA are expressed as mean ± SEM (*n = *3), **P<0.01. PPARγ activation did not significantly affect the PPARγ-PSF association. (B) To investigate the interaction between PSF and PPARγ, we analyzed their interaction in a mammalian two-hybrid assay in CV-1 cells. Treatment with rosiglitazone did not further stabilized PSF- PPARγ complex. Real-time PCR measurement of PPARγ mRNA expression under PSF knockdown conditions in HT-29 and DLD-1 cells (C) The relative PPARγ levels normalized to 18S rRNA are expressed as mean ± SEM (*n = *3), **P<0.01.
(EPS)

Table S1 **MALDI-TOF MS analysis for PSF siRNA transfected HT-29 and DLD-1 cells.** Analyses were performed using the AB SCIEX TOF/TOF™ 5800 System (AB SCIEX, Foster City, CA, USA). Protein identification was performed through ProteinPilot™ software (AB Sciex, Framingham, MA, USA) using the UniProt database. We identified 25 distinct proteins whose levels were significantly altered following PSF knockdown in both cell lines.
(XLS)
